# Probe into the pore structure of carbonated calcium silicate cement compacts by proton nuclear magnetic resonance relaxometry

**DOI:** 10.1039/d6ra04661d

**Published:** 2026-08-05

**Authors:** Zhikang Gao, Chen Li, Jinhao Zhang, Tiao Wang, Kaiming Peng, Zhengwu Jiang

**Affiliations:** a Key Laboratory of Advanced Civil Engineering Materials of Ministry of Education, School of Materials Science and Engineering, Tongji University Shanghai 201804 China lichen_0712@tongji.edu.cn jzhw@tongii.edu.cn; b Institute of Carbon Neutrality, Tongji University Shanghai 200092 China

## Abstract

Proton nuclear magnetic resonance relaxometry (^1^H NMR) has been widely used for characterizing the pore structure of Portland cement materials; however, its applicability to carbonated calcium silicate cement materials, an important CO_2_ capture technique applied in cementitious materials production, remains unresolved. This study systematically investigates the pore structures of carbonated calcium silicate cement compacts prepared from wollastonite, pseudowollastonite, γ-C_2_S and C_3_S using ^1^H NMR. It is observed that surface relaxivity and the relationship between magnetization signal intensity and sample mass differ significantly among carbonated samples and between situations when the samples are saturated with water and isopropanol (IPA). When fitting the curves of magnetization signal intensity *versus* sample mass, consistent slopes are observed for carbonated wollastonite and pseudowollastonite under both saturation situations. However, the slopes observed in carbonated γ-C_2_S and C_3_S under water-saturated conditions decrease with increasing carbonation time, whereas those under IPA-saturated conditions remain stable. ^1^H NMR can resolve nanopores (<10 nm) and capillary pores (10–1000 nm) in carbonated calcium silicate cements. The distributions of relaxation time evoluted during sample drying demonstrate that solvent removal occurs preferentially in capillary pores. Exchange of water by IPA treatment cannot remove all solvents from the samples. The findings contribute to elucidating the reliability of ^1^H NMR data-processing methods and provide a foundation for characterizing the pore structure of carbonated calcium silicate cement materials.

## Abstract 2

This work establishes a calibrated ^1^H NMR approach for decoding multiscale pore structures in carbonated calcium silicate cement materials under different solvent conditions.

## Graphical abstract

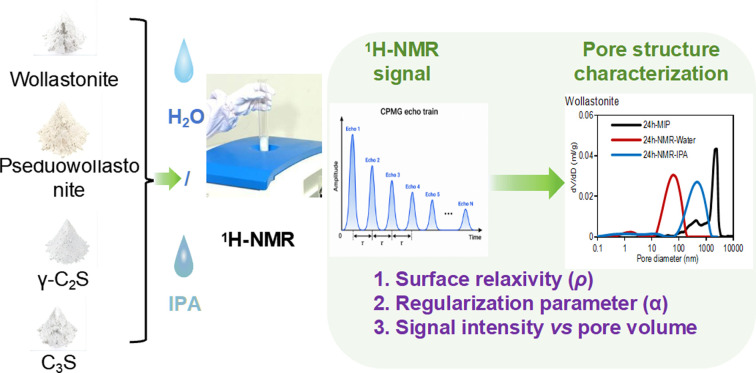

## 1 Introduction

The capture of CO_2_ from industrial flue gas for use in producing carbonated cementitious materials is considered a viable pathway to mitigate the global warming potential associated with the building sector.^1,2^ This technique typically employs calcium silicate minerals or industrial by-products containing these minerals as precursors for CO_2_ sequestration. From a stoichiometric perspective, each tonne of CaO can bind up to 0.78 tonnes of CO_2_.^3^ Globally, approximately 7 Gt of such by-products are generated annually, offering significant potential for CO_2_ sequestration and the production of carbonated cementitious materials.^4,5^

The CO_2_ sequestration capacity of calcium silicate materials is determined by both their intrinsic reactivity and, more critically, the pore structure, which governs the transport of reactive species and the buildup of carbonation products, thereby influencing the extent of carbonation.^6,7^ Previous studies have shown that carbonation efficiency is strongly influenced by the moisture state, pore structure, and formation of reaction products generated prior to the carbonation reaction.^8,9^ In carbonated cement materials comprising β-C_2_S, appropriate pre-conditioning can enhance CO_2_ uptake by facilitating CO_2_ diffusion and increasing reactive surface area, whereas the precipitations of carbonation products can hinder further diffusion of CO_2_ and calcium ions, thereby hindering the carbonation reaction.^9,10^ Similar transport limitations have also been observed in carbonated wollastonite, where carbonate precipitation and interfacial passivation restrict continued carbonation.^11^ These historic findings highlight the importance of pore structure that couples dynamically with the carbonation reaction and determines the mechanical and transport properties of carbonated calcium cement materials.

Proton nuclear magnetic resonance relaxometry (^1^H NMR) has recently emerged as a non-destructive, *in situ* technique for pore structure characterization in Portland cement materials.^12–15^ By combining the Carr–Purcell–Meiboom–Gill (CPMG) pulse sequence with inverse Laplace transform (ILT),^16,17^^1^H NMR tracks transverse relaxation time (*T*_2_) distributions that can be used in probing cement pore structures over a broad size range.^18^ Unlike traditional methods such as gas sorption or mercury intrusion, ^1^H NMR does not require sample drying and instead directly tracks the solvent signals within the pores, helping to preserve the intrinsic pore structure. This advantage is particularly important for cementitious materials because water is an integral part of the microstructure and its removal may irreversibly disturb the pore structure. In Portland cement and white cement, ^1^H NMR has been widely used to characterize gel pores and interlayer spaces in calcium silicate hydrate (C–S–H), including pores that are 1.5 and 4.1 nm in diameter.^17,19,20^ Growing evidence also shows that IPA exchange, an approach that has been extensively employed for cement hydration stoppage,^21,22^ can impact hydration products and consequently the measured pore structure.^23^

Existing ^1^H NMR pore quantification frameworks and related data-processing protocols were developed primarily for hydrated Portland cement and white cement, in which C–S–H is the main binding phase.^24–26^ In carbonated calcium silicate cement materials, however, the assemblage of phases may differ markedly. For instance, the carbonation of wollastonite and γ-C_2_S has been reported to generate calcium carbonate and decalcified silica gel.^27,28^ Studies on the carbonation of C_3_S and rankinite suggest that transient C–S–H may form initially and subsequently decompose into decalcified silica gel.^29^ These differences in reaction products may alter the chemistry of the pore wall, influencing the relaxation of protons in the pore solution. Consequently, parameters commonly used for Portland cement, such as surface relaxivity and the *T*_2_-to-pore-size conversion relationship, may not apply in carbonated calcium silicate cement materials.

To address these research gaps, this study investigates the applicability of ^1^H NMR relaxometry for use in characterizing the pore structure of carbonated calcium silicate cements prepared from natural wollastonite, pseudowollastonite, γ-C_2_S, and C_3_S. The carbonated materials were treated with two solvents, water and IPA, to assess the effect of solvent-exchange dewatering approach on the relaxation behavior of protons and the pore structure obtained. The impacts of regularization parameters (*α*), surface relaxivity (*ρ*), and the calibration of pore volume from magnetization signal intensity on the interpretation of ^1^H NMR data were highlighted. Furthermore, the evolution of the magnetization signal intensity during sample drying was tracked, and the behaviors of the water- and IPA-saturated samples to the drying procedures were compared. The findings contribute to clarifying the reliability of ^1^H NMR data-processing procedures, and provide a basis for quantifying the pore structure of carbonated calcium silicate cements.

## 2 Experiments

### 2.1 Raw materials

**Fig. 1:**
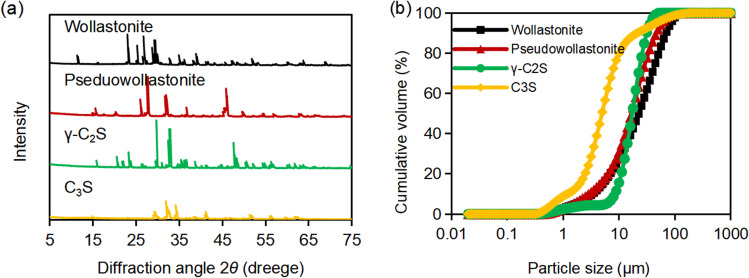
Raw materials: (a) XRD. (b) Particle size distribution.

Four precursors, namely, natural wollastonite, pseudowollastonite, γ-C_2_S, and C_3_S, were used for preparing carbonated calcium silicate cements. Pseudowollastonite and γ-C_2_S were synthesized from stoichiometric mixtures of reagent-grade CaCO_3_ and SiO_2_. The raw materials were first mixed according to the designed molar ratios. The mixture was heated to 900 °C at 10 °C min^−1^ and held for 1 h to ensure complete decomposition of CaCO_3_. The mixture was then further heated at 5 °C min^−1^ to 1420 °C (for pseudowollastonite) or 1400 °C (for γ-C_2_S) and held at the target temperature for 2 h, and then furnace-cooled to room temperature. The calcined products were subsequently crushed and sieved through a 200-mesh screen for later use. Natural wollastonite powder was commercially sourced from Lingshou Dehang Mineral Products Co., Ltd, and C_3_S powder was provided by Kunshan Huaqiao New Materials Co. These raw materials were sieved through a 200-mesh screen prior to storage to ensure the removal of large particles. As illustrated in the Fig. 1, the particle size distributions of natural wollastonite and pseudowollastonite are similar, whereas C_3_S exhibits the finest particle size distribution. The natural wollastonite contained a small amount of carbonate impurity, as indicated by the loss on ignition (LOI) and the calculated CaCO_3_ content in Table 1.

**Table 1:** Oxide composition of raw materials

	Wollastonite	Pseudowollastonite	γ-C_2_S	C_3_S
Na_2_O	–c	–c	–c	0.0
MgO	1.9	0.2	0.2	0.1
Al_2_O_3_	–c	–c	–c	–c
SiO_2_	42.6	49.9	33.8	26.3
CaO	42.3	44.4	62.1	70.3
Mn_3_O_4_	0.1	0.0	0.0	0.0
Fe_2_O_3_	0.3	0.3	0.0	0.1
LOI a	7.3	0.0	0.0	1.3
CaCO_3_^b^	16.6	0.0	0.0	0.0

a LOI (loss on ignition) is defined as the mass loss of the raw materials upon heating to 950 °C, the detailed calculation procedure is provided in the Supporting Information 1.

b CaCO_3_ content is quantified by the weight loss between 500–900 °C.

c “–” indicates a content lower than 0.001.

### 2.2 Sample preparation

Powders of calcium silicate materials were mixed with deionized water at a water-to-solid ratio of 0.12 and compacted into cylindrical samples (25 mm in diameter, approximately 6 mm in height) under an external pressure of 30 MPa. Two compacts were then placed in 300 mL reaction vessels and carbonated under a continuous flow of high-purity CO_2_ (99.999%) at 30 mL min^−1^. During carbonation, the vessels were maintained at 25 °C in a circulating water bath, while a relative humidity of approximately 90% was provided using a gas washing bottle. Carbonation durations were 1, 2, 6, 12, and 24 h.

After carbonation, samples were subjected to two solvent treatments. Considering both the drying procedure for carbonated calcium silicate materials and the solvent-saturation procedure for ^1^H NMR measurements, the samples were treated as follows: For water treatment, carbonated samples were placed in a vacuum desiccator, evacuated for 3 h, and then immersed in deionized water under vacuum for 24 h. For IPA treatment, carbonated samples were immersed in isopropanol for 48 h, then transferred to fresh IPA under vacuum for another 48 h, the samples were then weighed repeatedly until no appreciable change in mass was observed.

The samples were dried in a vacuum oven at 40 °C for approximately 48 h. Complete drying was operationally defined as the attainment of constant mass, with no appreciable mass change observed over a 12 h interval.

### 2.3 Operation of ^1^H NMR measurement

^1^H NMR measurements were performed using a PQ-001 spectrometer (Niumag Analytical Instrument Corporation, Suzhou, China) equipped with a 25 mm radio-frequency (RF) coil and operated at a magnetic field strength of 0.49 T with a resonance frequency of 21 MHz. The CPMG pulse sequence was used to acquire spin-echo decays. The echo time was set to 0.06 ms. The measurement level was set to 1, the repetition delay was 500 ms, the number of echoes was 10 000, and 128 scans were accumulated. The 90° and 180° pulse widths were set to 5.0 µs and 9.04 µs, respectively. Data acquisition and analysis were performed using software version V4.0.

### 2.4 Calibration of porosity and measurement of surface relaxivity (*ρ*)

Porosity of sample can be quantified by correlating the magnetization signal intensity of carbonated calcium silicate samples with corresponding mass loss. In ^1^H NMR studies of Portland cement, it is well established that the initial magnetization signal intensity of sample exhibits a strong linear relationship with water mass.^30^ In the present study, variations in sample mass were generated by progressively drying the samples in a forced-air oven at 40 °C. At each drying stage, the sample mass and magnetization signal intensity were measured using an analytical balance and ^1^H NMR spectrometer, respectively.

The surface relaxivity of sample can be obtained by fitting the derived equation under the conditions of short echo times and low solvent content. In ^1^H NMR measurements, the relaxation rate of pore solvent generally arises from two components: relaxation of water at the pore wall and within the pore space. Consequently, the relaxation time of the pore can be expressed by eqn (1).^14^1
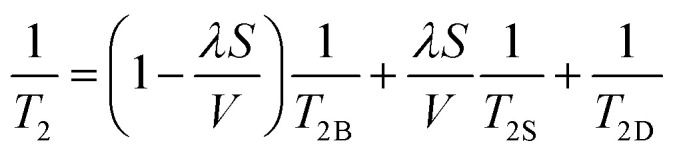
where *T*_2S_ represents the surface relaxation time, while *T*_2B_ denotes the transverse relaxation time of the bulk solvent within the pores; *T*_2D_ is the diffusion-induced relaxation time caused by the magnetic field gradient. *V* refers to the pore volume, *S* is the surface area of the pore walls, and *λ* is the thickness of the surface layer. Under solvent-saturated conditions, The bulk relaxation rate (1/*T*_2B_) is several orders of magnitude smaller than the surface relaxation rate (1/*T*_2S_) and can therefore be neglected.^18^ Moreover, by employing the CPMG sequence with a sufficiently short echo time, the contribution of diffusion-enhanced relaxation (1/*T*_2D_) can also be ignored. Therefore, pores of different sizes exhibit different relaxation times due to differences in their surface-to-volume ratio. The above eqn (1) can be simplified to eqn (2):2
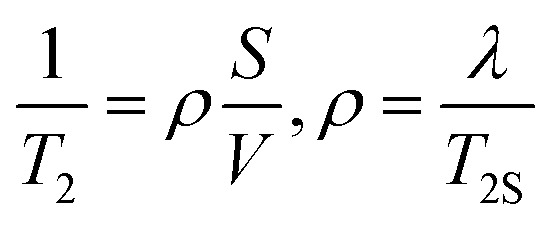


To calculate the surface relaxivity, both *T*_2S_ and *λ* are required. The initial magnetization signal intensity obtained from ^1^H NMR typically follows the mathematical relationship expressed in eqn (3):^14^3
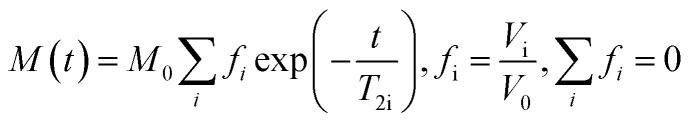
where *M*(*t*) represents the magnetization signal intensity at time *t*; *M*_0_ is the magnetization signal intensity at initial time; *V*_0_ denotes the total volume of the solvent; and *f*_*i*_ is the volumetric fraction, defined as the ratio of the solvent volume *V*_*i*_ in the *i*_-th_ discrete element to the total volume *V*_0_. A first-order Taylor expansion of eqn (3) about *t* = 0 yields eqn (4)4
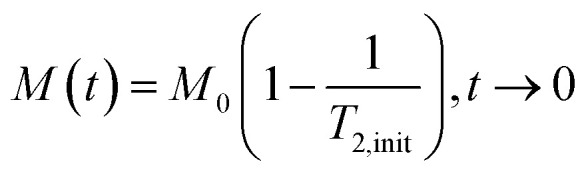
where *T*_2,init_ denotes the initial relaxation time of sample. When *t* approaches zero, *M*(*t*) exhibits a linear relationship with *t*. Measurements are performed by setting different echo times. Echo time is defined as the time interval from the center of the 90° pulse to the first magnetization signal intensity. *T*_2,init_ in this study is obtained by linear extrapolation from seven magnetization signal intensity values acquired at 10 µs intervals between 60 µs and 120 µs. *T*_2,init_ also satisfies eqn (5).^15^5
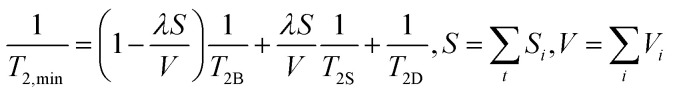
where *S*_*i*_ and *V*_*i*_ is the surface area and the volume of the *i*_-th_ discrete element. As the pore is progressively dried until only a monolayer of molecules covers the pore walls, the relaxation time is governed solely by water associated with the pore surface. The volume of the surface solvent layer equals the volume of the remaining pore solvent (*λ·S/V* = 1, *λ* = *D*), eqn (5) can be simplified to eqn (6).6
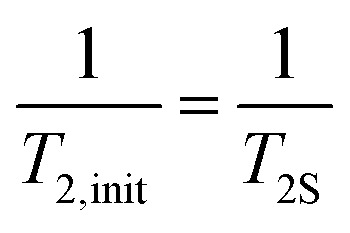
7
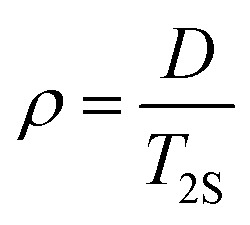
where *D* represents the molecular diameter of the solvent. It is established that the physical thickness of a monolayer of water molecules is approximately 0.3 nm,^31^ whereas the diameter of an isopropanol molecule is approximately 0.56 nm. Assuming a cylindrical pore geometry, this conversion can be expressed by eqn (8).^32^ After calculating the *ρ* according to eqn (7),^15^ this *ρ* was substituted into eqn (8) to convert the measured *T*_2_ values into equivalent pore size distributions based on the cylindrical pore model.8
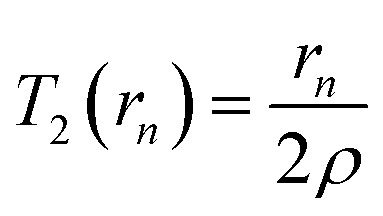
where *T*_2_(*r*_*n*_) represents the relaxation time corresponding to a pore with a radius of *r*_*n*_.

### 2.5 MIP

Mercury intrusion porosimetry (MIP) was performed on a Micromeritics AutoPore V 9620 porosimeter. The *Φ*25 mm × 6 mm samples were pretreated from IPA-saturated ones after testing by ^1^H NMR and further cut into 5 × 5 × 5 mm^3^ cubes. The porosity data were collected from 0.2 Pa to 60 MPa. The mercury contact angle and surface tension were set to 130° and 0.485 N m^−1^, respectively, and the pore-throat diameter was calculated using the Washburn equation.

## 3 Results

### 3.1 Measurement of surface relaxivity (*ρ*)

**Fig. 2:**
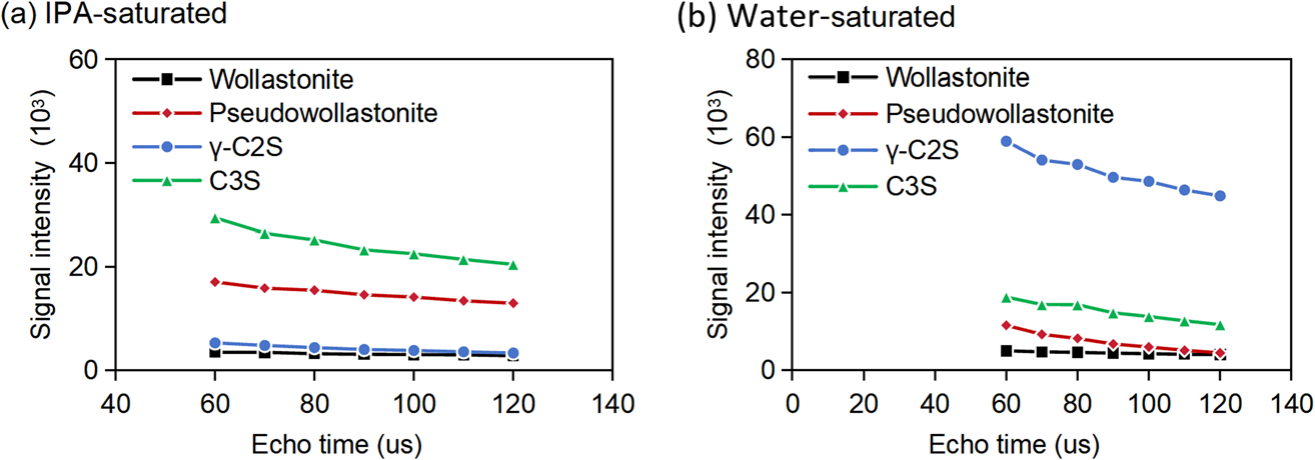
Magnetization signal intensity as a function of echo time in four carbonated calcium silicate materials with difference solvents: (a) water-saturated conditions, (b) IPA-saturated conditions.

To quantify the surface relaxivity of solvents in carbonated calcium silicate cement samples and calculate the radius of pores saturated by these solvents (eqn (2)), the intensity of magnetization (*M*(*t*)) acquired is plotted against echo time (*t*), see Fig. 2. Initial relaxation time (*T*_2,init_) is represented by the intersection of the plots with the horizontal axis (see eqn (4)), and the surface relaxivity can thus be acquired (eqn (7) and (8)), see Table 2.

**Table 2:** The surface relaxivity (*ρ*) of four carbonated calcium silicate materials

*ρ* (nm ms^−1^)	Wollastonite	Pseduowollastonite	γ-C_2_S	C_3_S
Water	0.82	1.72	1.18	1.27
IPA	1.50	1.45	2.45	1.83

The surface relaxivity is influenced by the solvent employed. For carbonated wollastonite, the surface relaxivity under IPA-saturated conditions is similar to that of carbonated pseudowollastonite. In contrast, the surface relaxivity of carbonated wollastonite under water-saturated conditions is only half of that measured in pseudowollastonite. A similar solvent-dependent trend is also found for γ-C_2_S and C_3_S. These results indicate that surface relaxivity needs to be calibrated when different calcium silicate minerals are used in carbonated cement materials production.

### 3.2 Calibration of porosity measurement

**Fig. 3:**
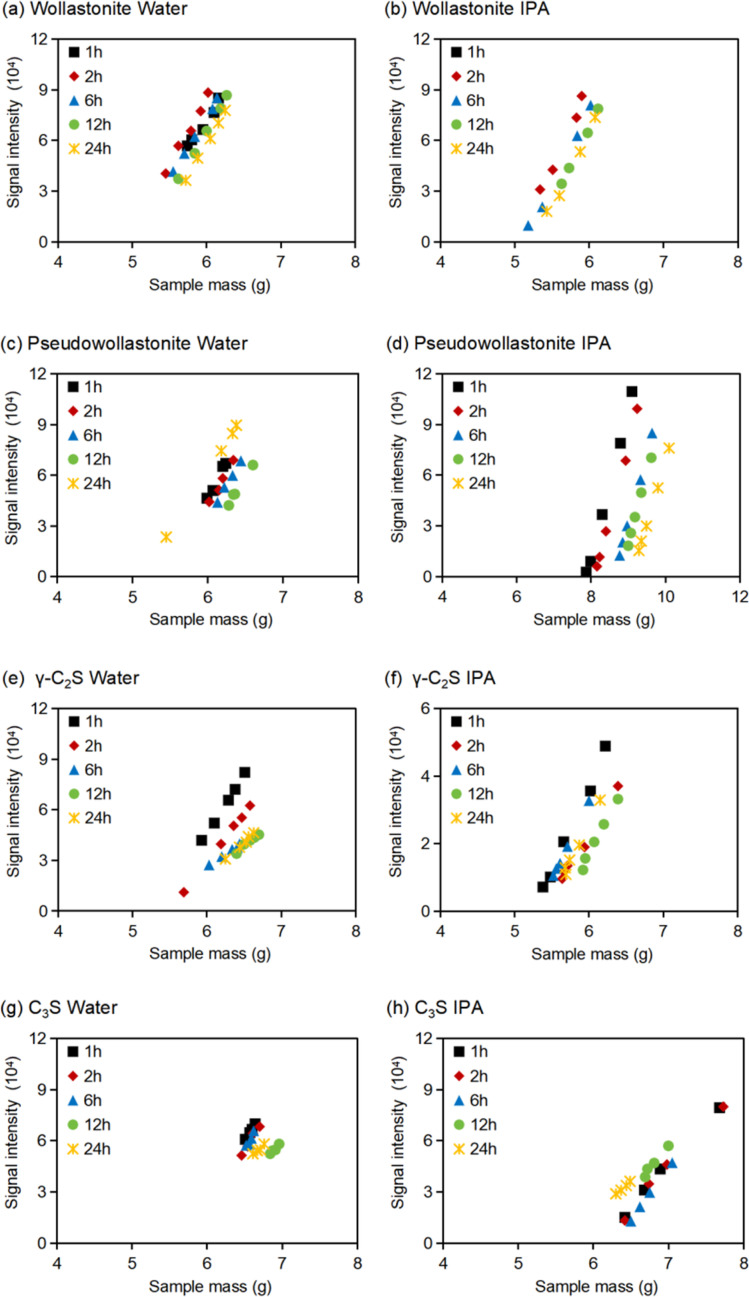
Quantitative ^1^H NMR results for carbonated calcium silicate materials: (a) water-saturated wollastonite, (b) IPA-saturated wollastonite, (c) water-saturated Pseudowollastonite, (d) IPA-saturated Pseudowollastonite, (e) water-saturated γ-C_2_S, (f) IPA-saturated γ-C_2_S, (g) water-saturated C_3_S, (h) IPA-saturated C_3_S

In hydrated Portland cement, porosity can be quantified using two calibration approaches: correlating the magnetization signal intensity with either the sample mass or the solvent mass.^14^ In the present study, porosity was quantified using the relationship between magnetization signal intensity and sample mass, while the alternative solvent-mass-based calibration method is presented in the SI 2. Applying this methodology to the carbonated calcium silicate materials, all carbonated samples exhibit a nearly linear relationship across various carbonation stages, see Fig. 3. The fitted curves of these linear relationship were compared across varying carbonation ages (Table 3).

**Table 3:** Fitted slope of the magnetization signal intensity–carbonated sample mass curves

Sample	Slope fitted at each carbonation time (10^4^/g)						
	1 h	2 h	6 h	12 h	24 h	Mean	SD
**Water-saturated samples**							
Wollastonite	7.21	7.74	7.75	7.56	7.70	7.59	0.227
Pseudowollastonite	8.79	7.87	7.71	7.23	7.03	7.73	0.686
γ-C_2_S	6.94	5.75	2.92	3.64	4.1	4.67	1.641
C_3_S	6.91	7.29	6.47	5.73	4.43	6.16	1.131

**IPA-saturated samples**							
Wollastonite	—	8.61	8.57	8.91	8.68	8.69	0.152
Pseudowollastonite	8.74	8.50	8.16	8.31	7.38	8.33	0.516
γ-C_2_S	4.90	3.56	4.92	4.31	4.45	4.48	0.555
C_3_S	5.01	4.96	6.18	5.46	3.82	5.43	0.860

For the water-saturated samples, the results reveal two distinctly behaviors: slopes of fitted curves (7.21–8.79) in carbonated wollastonite and pseudowollastonite maintain relatively stable throughout the entire carbonation process. The slope of fitted curves in carbonated γ-C_2_S and C_3_S decreases from 5.75–6.94 at early carbonation time (1–2 h) to 2.92–4.10 after prolonged carbonation (6–24 h). The slopes of the fitted curves in IPA-saturated sample remain strictly independent of the carbonation time for all minerals investigated here. However, the slopes of fitted curves in carbonated wollastonite and pseudowollastonite (7.38–8.74) are larger than those in carbonated γ-C_2_S and C_3_S (3.82–5.43). For the subsequent fitting of γ-C_2_S, the mean slope of the signal intensity–sample mass relationships obtained after 1 and 2 h of carbonation was used to quantify porosity.

The time-dependent variations in the slope of the fitted curves observed in water-saturated γ-C_2_S and C_3_S (Table 3) can be explained by the structural evolution of silica gel and the limitations of ^1^H NMR detection. In ^1^H NMR measurements, signals intensity from chemically bound or surface-adsorbed water may not be fully detected.^33^ The relaxation time of such water can be shorter than the interval after a radio-frequency pulse during which the same coil switches from transmitting to receiving, *i.e.*, the instrumental “dead time”.^33^ The microstructural configuration of silica gel in carbonated calcium silicate materials is scarce in literature. Here, the structure of the silica gel formed during carbonation is considered to be similar to Na_2_SiO_3_ gels. Such gels undergo continuous syneresis—characterized by network shrinkage and porhe gel into larger pores. Within these larger pores, the relaxation time of this water exceeds the instrumental dead time, transitioning this water from an “invisible” to a “detectable” state. The emergence of these previously undetected ^1^H NMR signals directly alters the cumulative magnetization signal intensity relative to the sample e-solvent expulsion—which drives the redistribution of the adsorbed water.^34^ During the drying phase, nanoscale silica gel particles (potentially < 10 nm in diameter) likely aggregate or undergo dehydroxylation,^28^ inducing significant structural rearrangements. Consequently, the water adsorbed or bound chemically on the gel may be expelled from the network structure of tmass, thereby modifying the resulting the slope of fitted curves.

### 3.3 Influence of regularization parameter (*α*) on *T*_2_ relaxation maps

In ^1^H NMR pore structure analysis, CPMG echo decays are typically processed *via* the ILT to obtain a quasi-continuous *T*_2_ distribution. However, because the ILT is inherently a mathematically ill-posed inverse problem, it is highly sensitive to experimental noise. Consequently, a regularization parameter (*α*) must be introduced to strike an optimal balance between data-fitting precision and solution smoothness. The selection of *α* profoundly influences the resolved *T*_2_ spectra and, by extension, the reliability of subsequent pore structure quantification.^35^

**Fig. 4:**
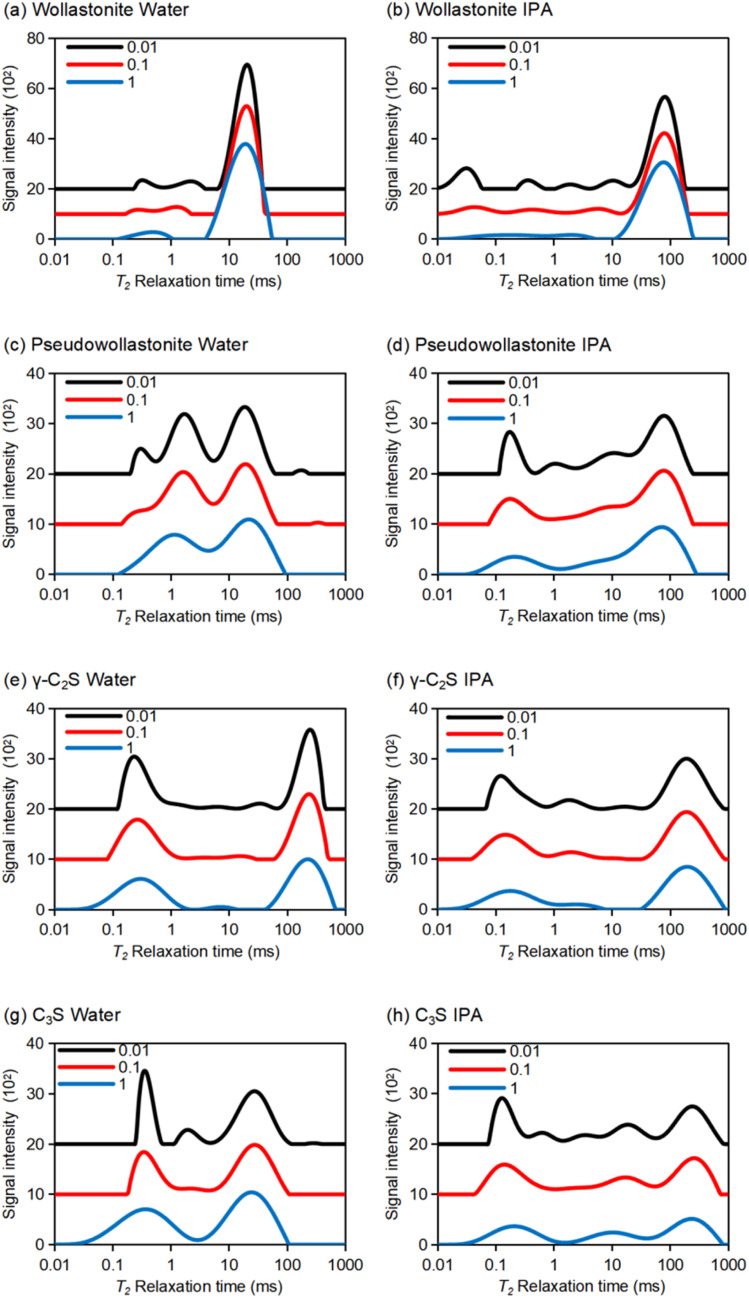
Inversion results of four calcium silicate materials after 24 h of carbonation using three different *α*: (a) water-saturated wollastonite, (b) IPA-saturated wollastonite, (c) water-saturated Pseudowollastonite, (d) IPA-saturated Pseudowollastonite, (e) water-saturated γ-C_2_S, (f) IPA-saturated γ-C_2_S, (g) water-saturated C_3_S, (h) IPA-saturated C_3_S.

Varying the *α* value leads to significant alterations in both the number and positions of the relaxation components (Fig. 4). At *α* = 0.01, the deconvolution of the *T*_2_ decay curves for water-saturated samples reveals three distinct relaxation components. For carbonated wollastonite, pseudowollastonite, and C_3_S, these components are centered at approximately 0.5, 5, and 50 ms, whereas for carbonated γ-C_2_S, these components are centered at roughly 0.5, 50, and 500 ms. When *α* is changed to 0.1, the intermediate components (near 5 ms) tend to merge with adjacent peaks, though this merging behavior varies depending on the materials. In carbonated wollastonite, pseudowollastonite, and γ-C_2_S, the 0.5 ms component broadens toward longer relaxation times, while in C_3_S, the 50 ms component shifts toward shorter relaxation times. At *α* = 1, the *T*_2_ map for all carbonated samples are simplified to predominantly two relaxation components. Although this higher *α* merges finely spaced adjacent peaks, it effectively mitigates noise-induced artifacts, and provide a robust and comparable result for analyzing the structural evolution across different materials.

The *T*_2_ map of the IPA-saturated samples exhibit a similar dependence on the *α* regarding the number and positions of the relaxation components. At *α* = 0.01, all carbonated samples display four distinct relaxation components; as *α* changes to 1, these relaxation components are reduced to three. This consolidation is dictated by the stronger smoothing penalty imposed by a larger *α*, which diminishes the mathematical ability to resolve closely spaced components.^36^ Conversely, while a smaller *α* enhances peak resolution, it is highly susceptible to under-smoothing and noise amplification. In carbonated wollastonite, the area of the relaxation component near 0.05 ms decreases markedly when *α* is changed from 0.01 to 0.1. Since the peak area strictly represents the magnetization signal intensity of a specific solvent population, this sharp reduction is anomalous. Previous studies on hydrated Portland cement paste have reported that modifying *α* primarily affects the width of the relaxation distribution, whereas the total area of intrinsic components remains largely conserved.^37^ Therefore, the strong *α*-dependence of the 0.05 ms component in the IPA-saturated wollastonite suggests that this peak is not a genuine physical pore population. Instead, it is likely a computational artifact arising from noise or inversion instability.

At an equivalent *α* value, the *T*_*2*_ spectra of the IPA-saturated samples consistently resolve one additional component (below 100 ms) compared to the water-saturated samples. This additional component can be fundamentally explained by the lower molecular diffusivity of IPA. According to the fast-exchange model,^38^ when solvent molecules diffuse rapidly among different pore environments during an echo interval, the corresponding relaxation components become indistinguishable and appear as a single volume-averaged relaxation time.^33^ Because water molecules possess a significantly higher molecular diffusivity than IPA in Portland cements.^39^ In the carbonated calcium silicate materials, the more sluggish diffusivity of IPA restricts solvent exchange among pore. Consequently, components that appear as a single merged component in the water-saturated state can be successfully resolved as separate, distinct relaxation components when probed with IPA.

### 3.4 Equivalent pore size distributions compared with MIP

**Fig. 5:**
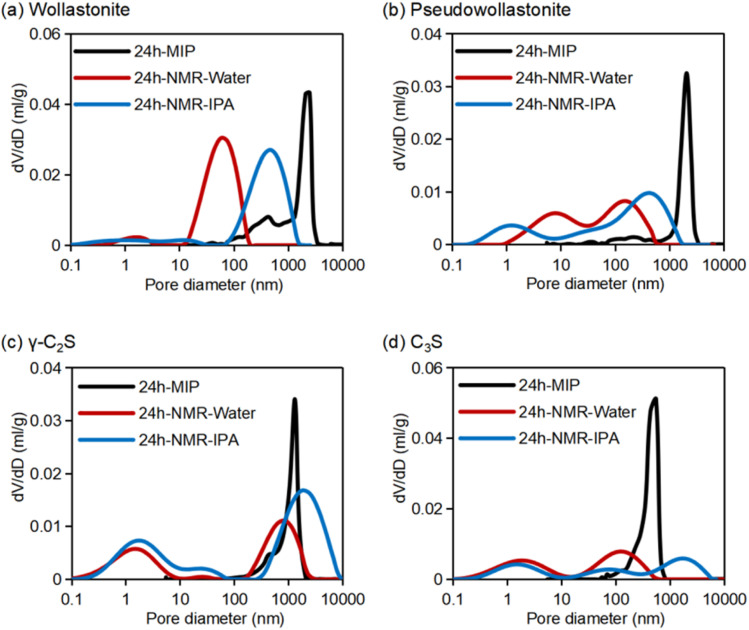
Equivalent pore size distribution curves obtained from ^1^H NMR in water-saturated and IPA-saturated conditions and MIP. For carbonated γ-C_2_S and C_3_S, the porosity was calculated using calibration results from two carbonation stages (1 h, 2 h) s*ee* support information 4: (a) Wollastonite, (b) Pseudowollastonite, (c) γ-C_2_S, (d) C_3_S.

To facilitate direct structural comparison among the carbonated calcium silicate materials, the *T*_2_ distributions obtained using *α* = 1 were converted into equivalent pore-size distributions, as shown in Fig. 5. The *T*_2_ distributions and the corresponding converted pore sizes reveal two distinct pore populations: nanopores (1–10 nm; *T*_2_ approx 0.1–1 ms) and capillary pores (10–1000 nm; *T*_*2*_ approx 10–1000 ms),No mesopor (2–50 nm) was observed in the MIP results. We therefore consider that the nanopores are mainly associated with the silica gel formed during carbonation, whereas the capillary pores primarily originate from the interparticle voids formed by particle packing.

For wollastonite, γ-C_2_S, and C_3_S, changing the saturated solvent from water to IPA did not produce an obvious shift in the characteristic nanopore size, suggesting that IPA exchange had a limited influence on the characteristic dimensions of these nanopore. In contrast, the nanopore component of pseudowollastonite shifted toward a smaller equivalent pore size after IPA exchange, which may indicate partial contraction or rearrangement of its nanoporous structure.

The capillary pore sizes of all four materials increased after IPA exchange. As discussed in Section 3.2, this change cannot be directly attributed to drying-induced shrinkage of the silica gel, as is commonly considered for hydrated Portland cement. A more plausible explanation is that the relative contributions of different pore domains to the measured relaxation response changed after solvent exchange. According to the fast-exchange model,^38^ the observed relaxation response represents a population-weighted average of the solvent distributed within an interconnected pore network. The MIP results indicate the coexistence of two characteristic capillary pore in wollastonite and pseudowollastonite. Because molecular diffusion and exchange are slower for IPA than for water,^40^ solvent confined in some smaller pore domains may not exchange sufficiently rapidly with the rest of the pore network within the echo time. These domains may consequently be resolved as separate shorter *T*_2_ components, while the relative contribution of the larger capillary pores to the remaining capillary pore component increases. This redistribution of signal contributions would shift the population-averaged *T*_2_ toward longer relaxation times, thereby producing a larger apparent equivalent capillary-pore size without necessarily indicating actual pore coarsening.

For γ-C_2_S and C_3_S, the equivalent pore sizes obtained by MIP were smaller than those derived from ^1^H NMR under IPA-saturated conditions. It should be noted that MIP primarily reflects the narrow pore entrances or pore throats controlling mercury intrusion and is therefore strongly affected by pore connectivity, constrictions, and ink-bottle effects.^41^^1^H NMR probes the volumetric relaxation response of the pore-saturated solvent and therefore more closely reflects the relaxation environment within the pore bodies. Consequently, for pore networks in which relatively large pore bodies are connected through narrower throats, the equivalent pore sizes derived from ^1^H NMR may be larger than the pore-entry sizes measured by MIP.

In addition, the pore-size distributions of ^1^H NMR were obtained by converting the *T*_2_ distributions using an idealized cylindrical pore model. Because the actual pores in the carbonated calcium silicate materials are irregular and cannot be represented as perfect cylinders, unavoidable deviations may exist between the model-derived equivalent pore sizes and the actual pore dimensions. A cylindrical pore model was adopted in this study. In the data analysis, alternative pore geometries would change the surface-to-volume ratio (S/V) in eqn (2), thereby altering the proportional relationship between *T*_2_ and the equivalent pore size.

**Fig. 6:**
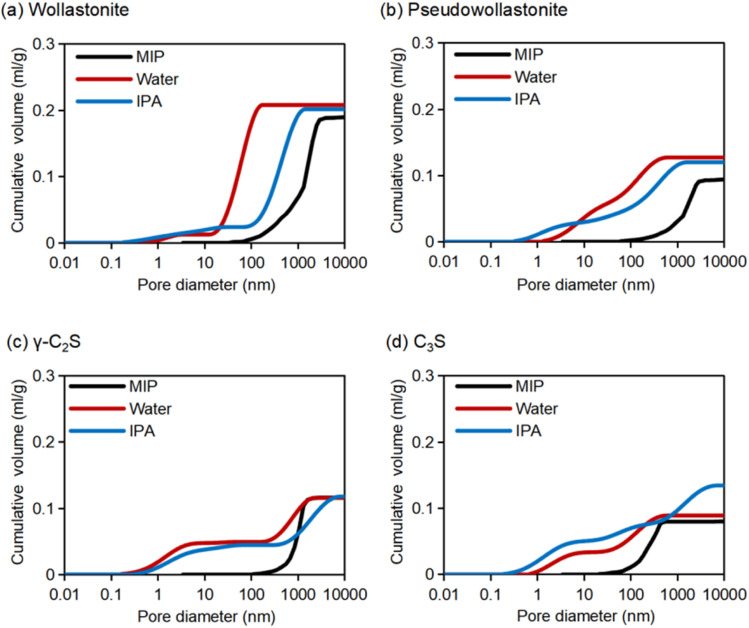
Cumulative pore size distribution curves obtained from ^1^H NMR in water-saturated and IPA-saturated conditions and MIP: (a) Wollastonite, (b) Pseudowollastonite, (c) γ-C_2_S, (d) C_3_S.

Compared with porosity obtained by MIP, the capillary pore sizes of water-saturated samples obtained by ^1^H NMR are smaller than those measured by MIP across all carbonated materials (Fig. 6). For wollastonite and pseudowollastonite, the porosities measured under water-saturated and IPA-exchanged conditions were comparable, and the capillary pore volumes determined by ^1^H NMR agreed well with those measured by MIP, suggesting that most of the capillary pore volume was captured under both conditioning states. For γ-C_2_S and C_3_S, the total porosities obtained by MIP were also comparable to those measured by ^1^H NMR. Because MIP has limited accessibility to the finest nanopores, this apparent agreement may partly result from drying-induced shrinkage or collapse of the nanoporous structure before mercury intrusion. For C_3_S, the interpretation is further complicated by its hydration activity, because water saturation may promote additional hydration and alter the pore structure measured by ^1^H NMR.

It should be noted that the data-point density of the ^1^H NMR result is substantially higher than that of the MIP results, particularly in the vicinity of the distribution peaks. Consequently, a direct comparison based on peak height is not reliable because the apparent peak intensity is affected by the sampling density and data-processing method. Therefore, the distribution curves are used only to qualitatively compare the pore-size ranges and overall distribution characteristics. For improved clarity, the corresponding scatter plots showing the original data points are provided in the SI 5.

### 3.5 Removal of water and IPA during drying

**Fig. 7:**
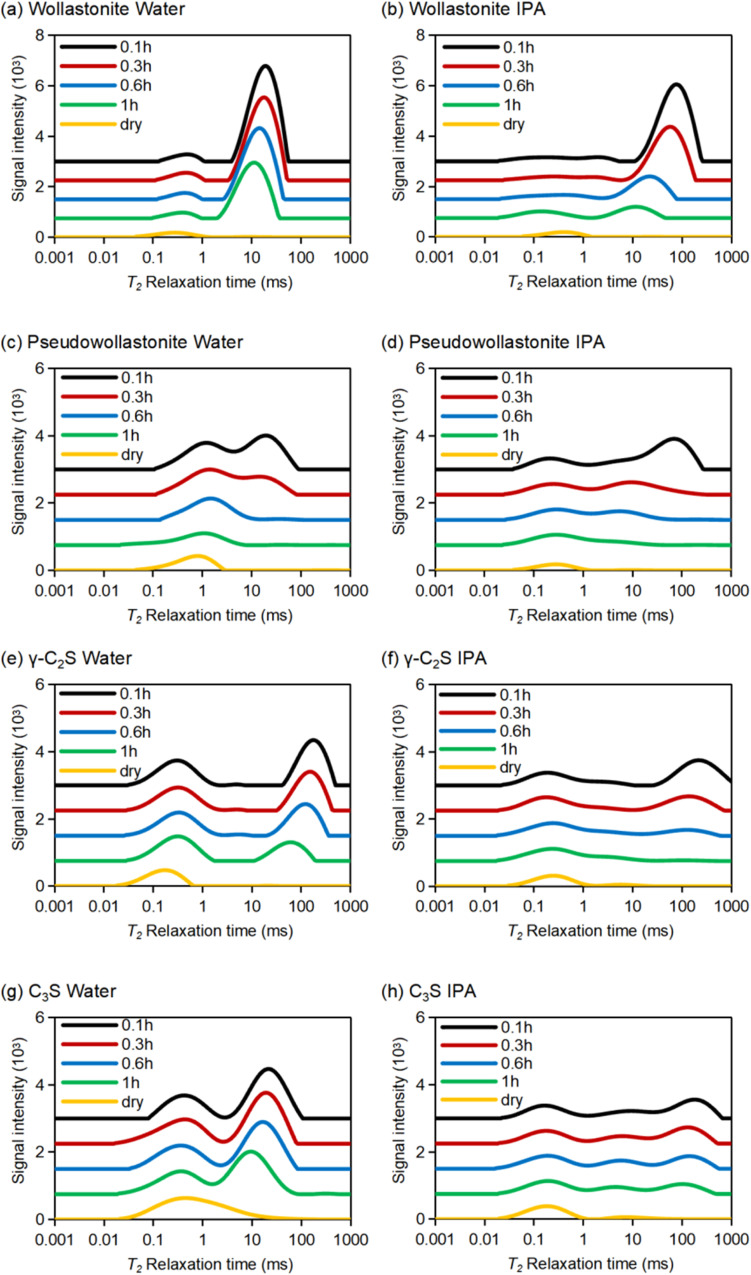
*T*_2_ distribution maps of four carbonated calcium silicate materials at carbonated 24 h during drying for 0.1, 0.3, 0.6, and 1.0 h, and after drying to constant mass. “Dry” means dried sample: (a) water-saturated wollastonite, (b) IPA-saturated wollastonite, (c) water-saturated Pseudowollastonite, (d) IPA-saturated Pseudowollastonite, (e) water-saturated γ-C_2_S, (f) IPA-saturated γ-C_2_S, (g) water-saturated C_3_S, (h) IPA-saturated C_3_S. The quantitative water contents associated with the different pore-size ranges are provided in the SI 6.

To dynamically track solvent migration and spatial redistribution within the pore network during drying, continuous *T*_2_ distribution maps were acquired throughout the drying process using a regularization parameter of *α* = 1 (Fig. 7). As drying proceeds, the relaxation component corresponding to capillary pores (centered near 100 ms) progressively diminishes in magnetization signal intensity and shifts toward shorter relaxation times across all carbonated samples. The component of nanopore (centered at approx 0.5 ms) remains positionally stable during the initial 1 h of drying.

The magnetization signal intensity of nanopore-scale component remained nearly unchanged during the initial drying process. After IPA exchange and subsequent complete drying, the signal contributed from the nanopore-scale component decreased substantially, while its *T*_2_ position showed no obvious shift. This suggests that the amount of mobile or detectable hydrogen-containing species within the nanopores was reduced, whereas the local confinement environment of the remaining species was not significantly altered. Given that the intrinsic *T*_2_ of confined IPA theoretically exceeds that of confined water,^42,43^ the positional stability of this component (at 0.5 ms) implies that this signal may originate from bound water within the nanopores, rather than from the IPA. IPA exchange—while capable of modifying and even coarsening the pore structure—often results in an incomplete replacement of the strongly confined pore water.^39,42^ But the relaxation time is also affected by surface relaxivity, pore-wall chemistry, confinement, and molecular exchange. Therefore, the similar peak position may also result from IPA-induced changes in the pore structure or specific interactions between IPA and the pore surfaces.

## 4 Conclusions

This study investigates the applicability of ^1^H NMR for use in characterizing the multiscale pore structure of carbonated calcium silicate cement compacts, and use this technique to track the removal of solvents from pores during the drying processes of these cements. The main conclusions are as follows:

1. When quantifying the pore structure of carbonated calcium silicate cements by ^1^H NMR, different surface relaxivity were observed across the four calcium silicate precursors (wollastonite, pseudowollastonite, γ-C_2_S and C_3_S) and when the carbonated samples are saturated with water and IPA.

2. The slopes of the fitted curves between magnetization signal intensity and sample mass differ among carbonated calcium silicate materials. Consistent slopes are observed for carbonated wollastonite and pseudowollastonite under both saturation conditions. The slopes for water-saturated γ-C_2_S and C_3_S decrease with increasing carbonation time, yet remain stable under IPA saturated conditions. Despite these variations of slopes, directly correlating the signal intensity with sample mass constitutes a good methodology for porosity quantification.

3. The ^1^H NMR effectively resolves two distinct relaxation time components within the carbonated calcium silicate materials: a relaxation component at 0.1–1 ms corresponding to nanopores (<10 nm), and another relaxation component at 10–1000 ms corresponding to capillary pores (10–1000 nm). The nanopores cannot be well detected by MIP. The capillary pore sizes measured by ^1^H NMR differ from those observed *via* MIP and are primarily attributable to their distinct mechanisms by which the pores are arrested.

4. The relaxation component associated with nanopores remained nearly unchanged during drying for the water-saturated samples, whilst the intensity of this relaxation component decreased when the samples are saturated by IPA. This observation indicates that IPA exchange may alter the accessible nanopore volume without influencing their sizes.

## Author contributions

Zhikang Gao: methodology, investigation, formal analysis, visualization, writing – original draft. Chen Li: conceptualization, formal analysis, data curation, writing – original draft, writing – review and editing, supervision, funding acquisition, project administration. Jinhao Zhang: methodology, investigation. Tiao Wang: formal analysis. Kaiming Peng: writing – review and editing. Zhengwu Jiang: supervision, funding acquisition, project administration.

## Conflicts of interest

All authors declare that they have no conflicts of interest.

## Supplementary Material

RA-OLF-D6RA04661D-s001

## Data Availability

The data that support the findings of this study are available from the corresponding author upon reasonable request. Supplementary information (SI) is available. See DOI: https://doi.org/10.1039/d6ra04661d.
