# Supplementary material for: Probe into the pore structure of carbonated calcium silicate cement compacts by proton nuclear magnetic resonance relaxometry
Source: RSC Adv. 2026 Aug 5;16(39):45028–39. doi: 10.1039/d6ra04661d (PMC13440587; doi:10.1039/d6ra04661d)
Supplement: Supplementary file 1 [file RA-016-D6RA04661D-s001.pdf]

Supplementary Information (SI) for RSC Advances.
This journal is © The Royal Society of Chemistry 2026

Supplementary Information

Probe into the pore structure of carbonated calcium silicate cement compacts by proton nuclear magnetic resonance relaxometry

Zhikang Gao [a], Chen Li [a,b*], Jinhao Zhang [a],Tiao Wang [a], Kaiming Peng [b], Zhengwu Jiang [a,*]

[a] Key Laboratory of Advanced Civil Engineering Materials of Ministry of Education, School of Materials Science and Engineering, Tongji University, Shanghai, 201804, China

[b] Institute of Carbon Neutrality, Tongji University, Shanghai 200092, China

[*] Corresponding author: lichen_0712@tongji.edu.cn (C. Li); jzhw@tongii.edu.cn (Z. Jiang)

1 TG-DTG analysis of raw materials and calculation of loss on ignition (LOI)

The loss-of-ignition at 1000 °C was tested using a TA TGA 550 thermal analyzer. The results are shown in figure 1.

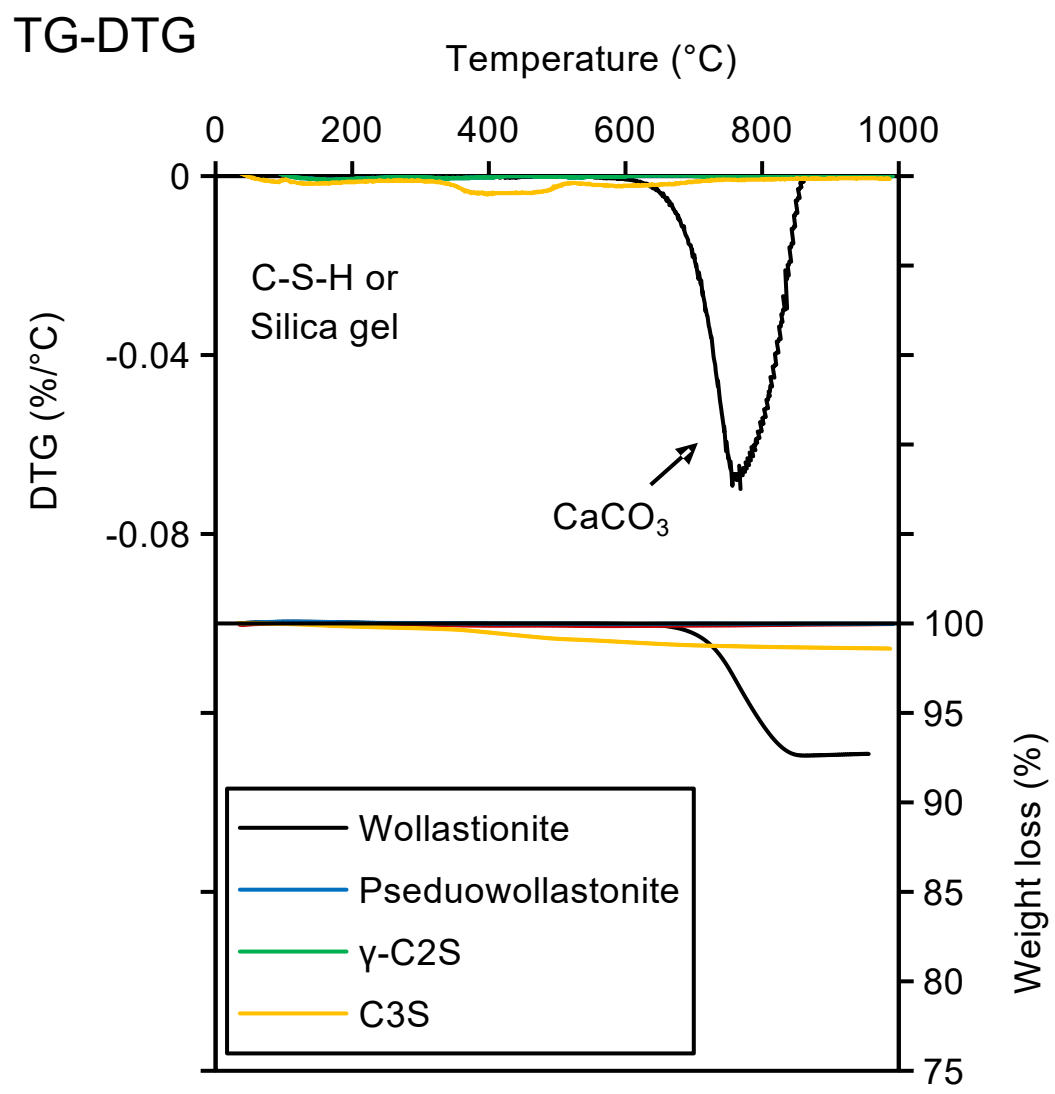

Figure 1. TG–DTG curves of the four raw materials.

In the original manuscript, the LOI values reported in Table 1 were calculated according to Eq. (1).

$$LOI = (M_{25} - M_{950})/M_{25} \qquad (1)$$

$$W_{CaCO3,\ measured} = (M_{500} - M_{950}) \times \frac{100}{44} \qquad (2)$$

$$W_{CaCO3,\ normalized} = W_{CaCO3,\ measured} \times \frac{(1 - LOI) \times M_{25}}{M_{950}} \qquad (3)$$

Where $W_{CaCO3,measured}$ and $W_{CaCO3,normalized}$ denote the $CaCO_3$ content measured in the sample and the $CaCO_3$ content normalized to 100g of raw material, respectively; M25, M500, and M950 represent the sample masses measured at 25°C, 500°C, and 950°C. LOI denotes the loss on ignition of the raw material determined by TG analysis.

2 Fitting results for the relationship between ^{1}H-NMR signal intensity and water mass in the samples

In reference 1, porosity was quantified by correlating the magnetization signal intensity with the mass of the solvent contained within the pores. This solvent mass is determined indirectly by calculating the mass difference between the test sample and

its fully dried state (constant mass). When this approach is applied to carbonated calcium silicate materials, the fitted curves of the magnetization signal intensity with solvent mass varies depending on the solvent for saturation and material (*Figure 2*). Under water-saturated conditions, the coefficients of determination (R^2) for fitted curves difference considerably (*Table 1*). While carbonated wollastonite maintains a robust linear fit ($R^2 > 0.95$), the other water-saturated carbonated samples exhibit substantially lower fitting reliability ($R^2 < 0.90$). In contrast, when saturated with IPA, all carbonated samples consistently demonstrate high fitting reliability ($R^2 > 0.95$). Compared this subtraction-based approach, directly establishing the fitted curves between the magnetization signal intensity and sample mass constitutes a more robust and appropriate methodology for quantifying the porosity of carbonated materials.

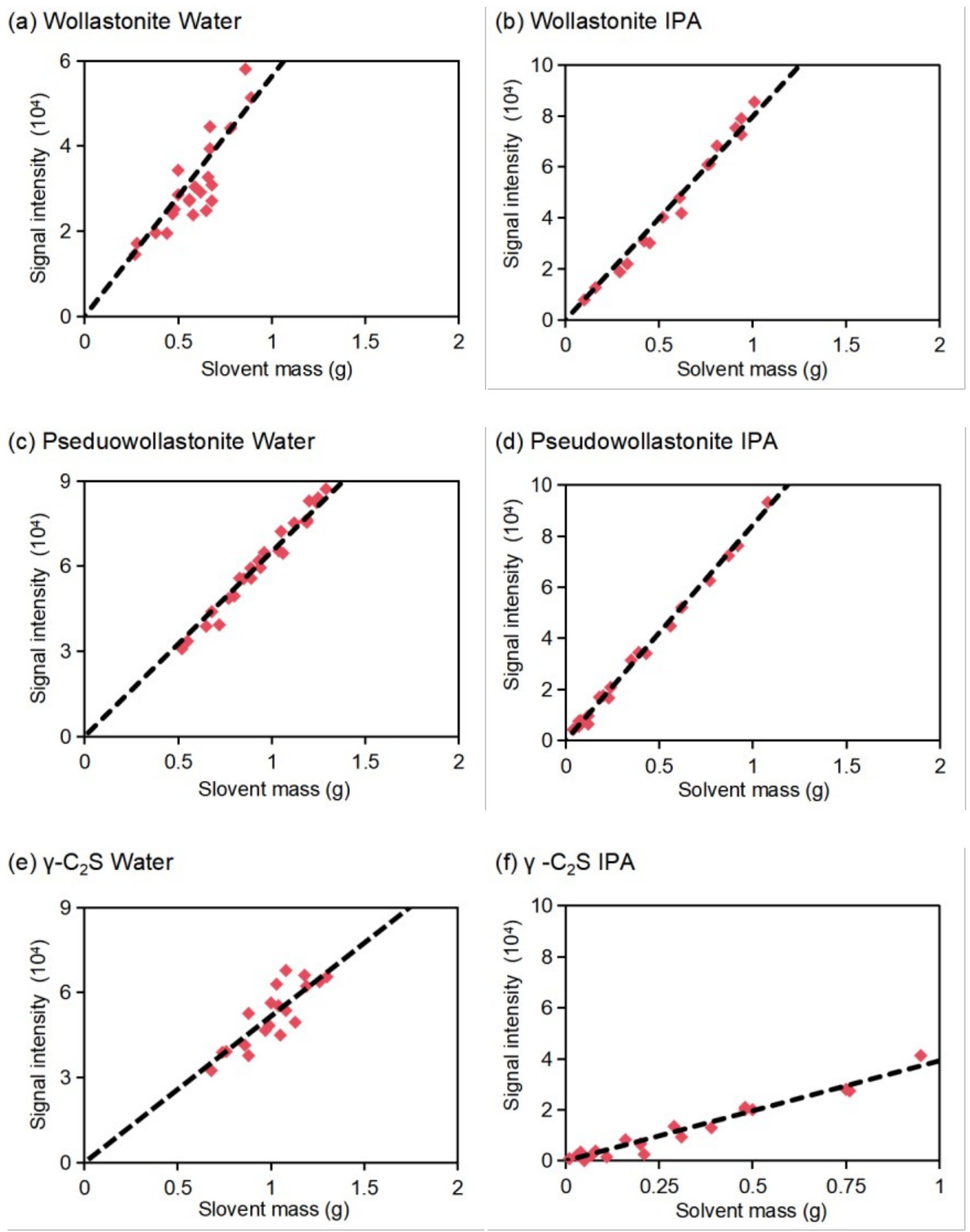

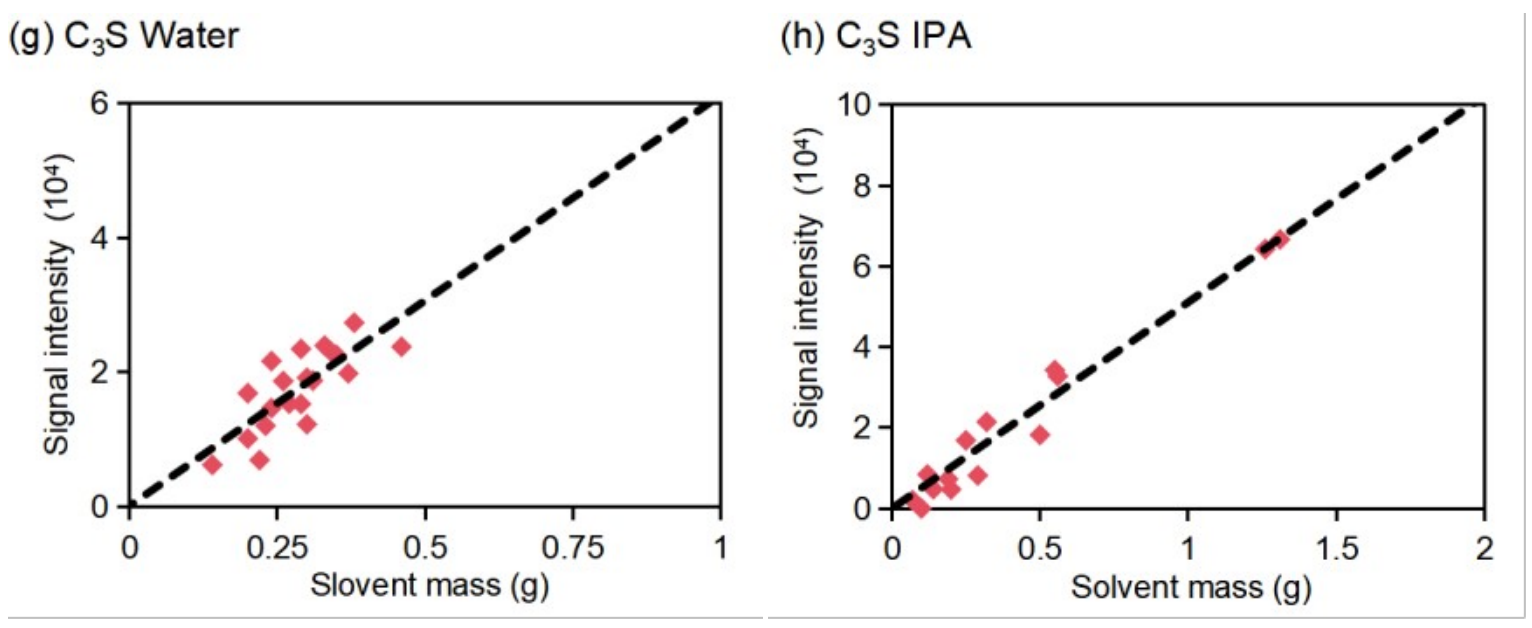

Figure 2. Quantitative magnetization signal intensity with mass of solvent in sample:(a) water-saturated wollastonite, (b) IPA-saturated wollastonite, (c) water-saturated Pseudowollastonite, (d) IPA-saturated Pseudowollastonite, (e) water-saturated γ-C_2S, (f) IPA-saturated γ-C_2S, (g) water-saturated C_3S, (h) IPA-saturated C_3S

Table 1 Fitted slope of the magnetization signal intensity–solvent mass curves.

Sample	Slope fitted by all carbonated samples (10^4/g)	
	Slope	R^2
Water		
Wollastonite	7.18	0.97
Pseudowollastonite	6.07	0.74
γ-C_2S	5.37	0.84
C_3S	7.26	0.88
IPA		
Wollastonite	8.61	0.98
Pseudowollastonite	8.44	0.99
γ-C_2S	4.05	0.96
C_3S	5.11	0.95

3 Repeatability test results for the relationship between signal intensity and sample mass in different carbonated calcium silicate materials

For each material, selected carbonation ages were measured repeatedly, or additional specimens carbonated for the same duration were tested. As shown in Figure 3, the linear relationships obtained from the repeated measurements were nearly parallel for each material, indicating good reproducibility and consistency of the measurement and calibration procedures. In addition, it should be noted that each data point represents the average obtained from 128 scans.

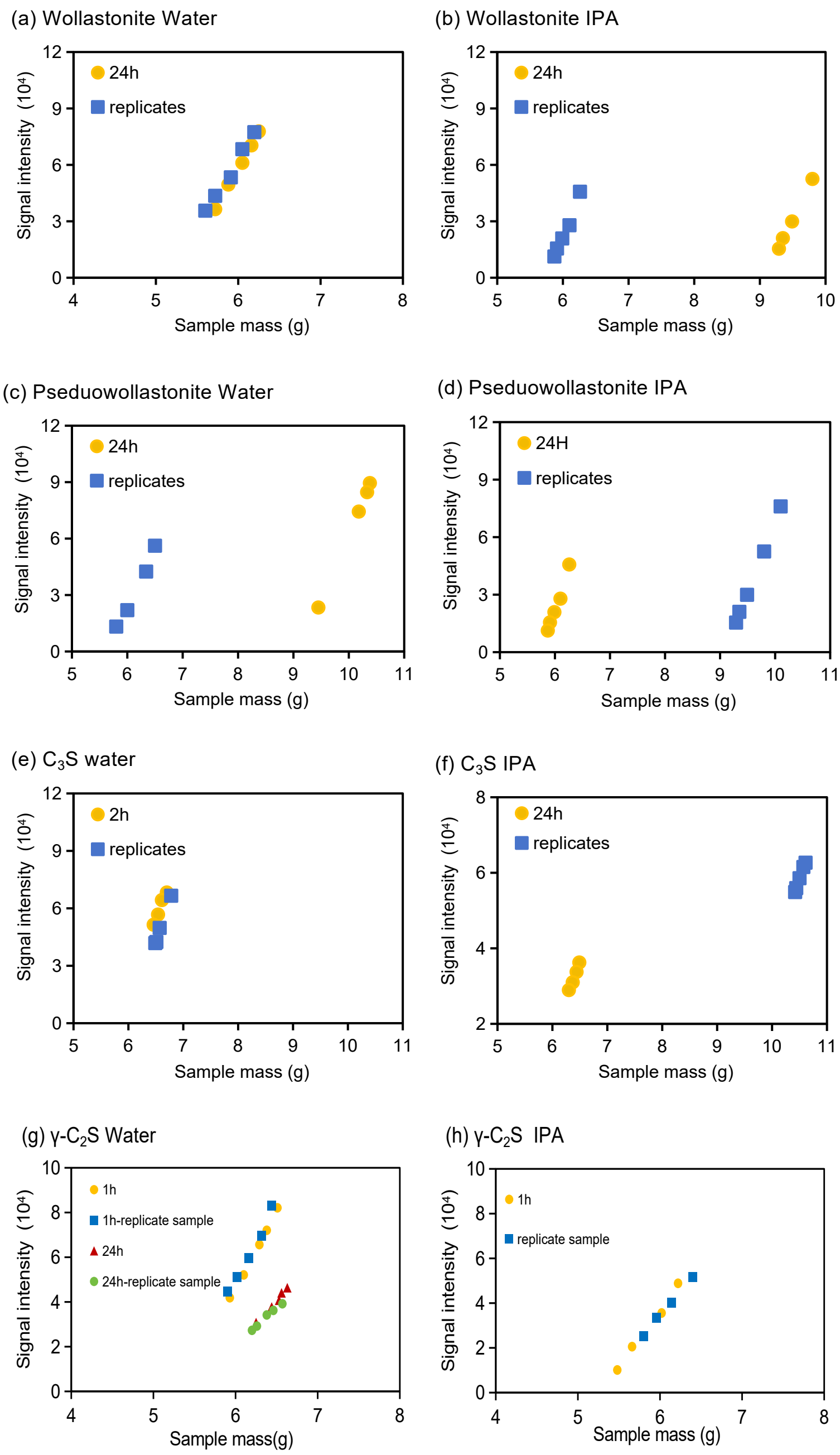

Figure 3. Results of the repeatability tests:(a) water-saturated wollastonite, (b) IPA-saturated wollastonite, (c) water-saturated Pseudowollastonite, (d) IPA-saturated Pseudowollastonite, (e) water-saturated C_3S, (f) IPA-saturated C_3S, (g) water-saturated γ-C_2S, (h) IPA-saturated γ-C_2S

4 Selection criteria and related tests for the γ-C_2S fitting curves

The fitted curves of the water-saturated γ-C_2S samples demonstrated excellent reproducibility (*Figure 4*). These samples were further dried to a constant mass. In the γ-C_2S carbonated for 1 h, the recorded datum representing dried sample aligns perfectly

with its established fitted curves. In the γ-C_2S carbonated for 24 h, however, this datum exhibits a distinct deviation from the fitted curves. Such an observation can be indirect evidence supporting the altering of silica gel structure that impacts the magnetization signal intensity-mass relationship. It is likely that the fitted curves derived from early carbonation stages provides a significantly more reliable and accurate baseline for the quantitative evaluation of porosity.

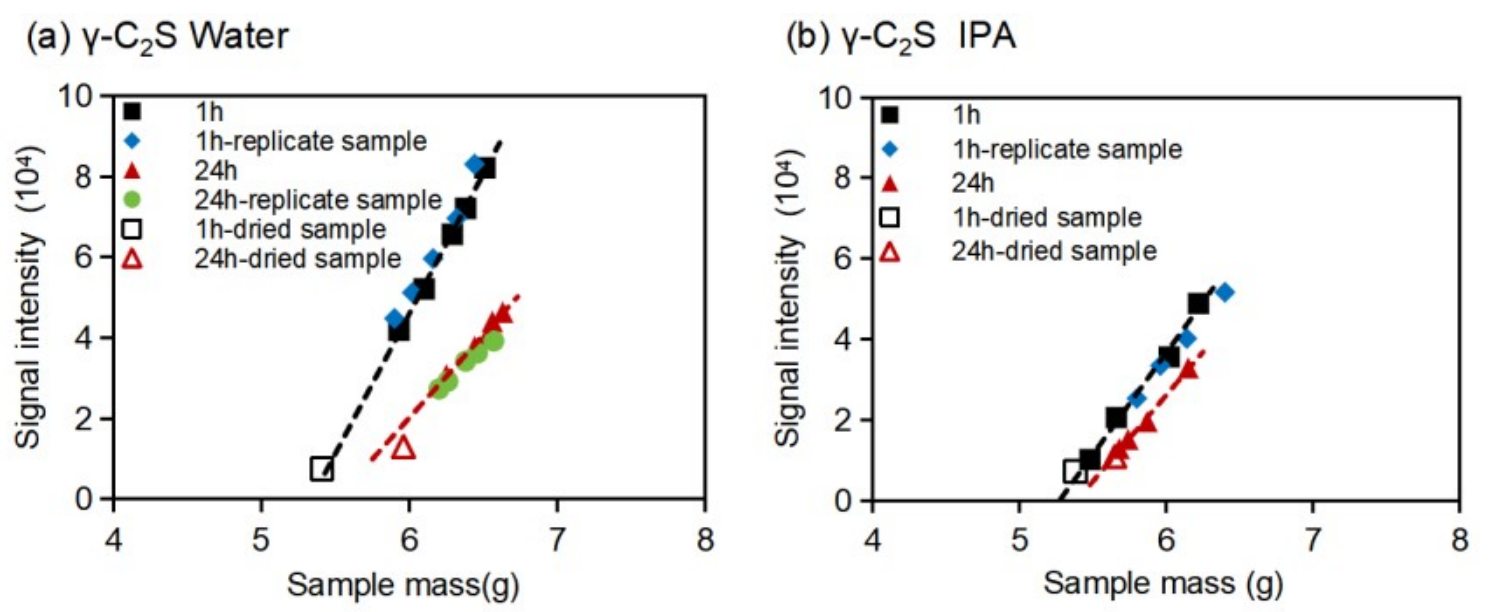

Figure 4. ^{1}H-NMR repeatability test of γ-C_2S and the relationship between dry mass and fitted line position. The black and red line is the fitted curves of magnetization signal intensity versus sample mass during drying (excluding the dried sample). (a) water-saturated γ-C_2S, (b) IPA-saturated γ-C_2S

For carbonated γ-C_2S samples that were saturated with IPA, data points corresponding to the sample that was dried to constant mass align with the fitted curves, demonstrating excellent reproducibility. Nevertheless, the overall IPA magnetization signal intensity in γ-C_2S is significantly lower than that observed in wollastonite. While the exact mechanism remains to be fully elucidated.

5 Point–line plots comparing the low-field NMR and MIP results

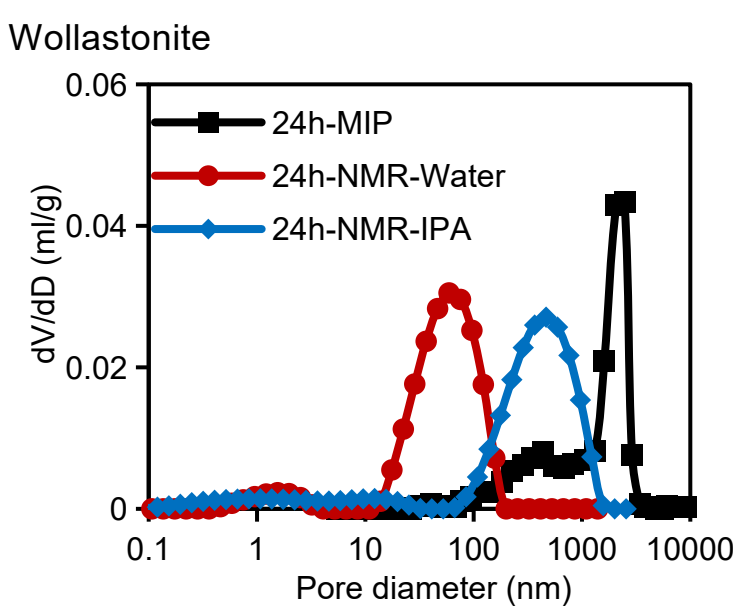

Figure 5. Point–line plots comparing the MIP and ^{1}H-NMR results for wollastonite.

6 Changes in Solvent Content across Different Pore-Size Ranges（Figger 7）

Table 2 Signal intensity of solvent in nanopore and capillary pores

Sample	Signal intensity (10^4/g)					
	Scale	0.1 h	0.3 h	0.6 h	1 h	Dry sample
Water-saturated samples						
Wollastonite	nanopore	4.73	5.29	4.08	3.96	5.44
	capillary pores	73.08	65.08	57.02	45.58	
Pseudowollastonite	nanopore	26.91	28.72	21.47	13.03	13.03
	capillary pores	29.30	13.76	0.49	0.20	
γ-C_2S	nanopore	19.15	18.90	18.54	19.24	13.69
	capillary pores	24.93	21.87	19.22	11.55	
C_3S	nanopore	14.57	20.80	19.42	18.04	35.54
	capillary pores	34.84	35.05	33.79	35.42	
IPA-saturated samples						
Wollastonite	nanopore	8.70	6.88	6.83	7.88	5.49
	capillary pores	64.96	46.44	20.52	10.19	
Pseudowollastonite	nanopore	10.80	11.91	11.78	11.79	5.43
	capillary pores	34.94	16.15	9.09	3.74	
γ-C_2S	nanopore	11.38	12.15	12.67	12.11	10.86
	capillary pores	21.78	13.56	7.81	3.51	
C_3S	nanopore	12.30	12.55	13.72	13.44	14.31
	capillary pores	23.94	21.20	18.27	15.50	

1. C. S. Zhou, F. Z. Ren, Z. D. Wang, W. Chen and W. Wang, Cement Concrete Res, 2017, 100, 373–384.
